# The Clinical Utility of a 7-Gene Biosignature on Radiation Therapy Decision Making in Patients with Ductal Carcinoma In Situ Following Breast-Conserving Surgery: An Updated Analysis of the DCISionRT^®^ PREDICT Study

**DOI:** 10.1245/s10434-024-15566-5

**Published:** 2024-06-25

**Authors:** Chirag Shah, Pat Whitworth, Frank A. Vicini, Steven Narod, Naamit Gerber, Sachin R. Jhawar, Tari A. King, Elizabeth A. Mittendorf, Shawna C. Willey, Rachel Rabinovich, Linsey Gold, Eric Brown, Anushka Patel, John Vargo, Parul N. Barry, David Rock, Neil Friedman, Gauri Bedi, Sandra Templeton, Sheree Brown, Robert Gabordi, Lee Riley, Lucy Lee, Paul Baron, Lonika Majithia, Kristina L. Mirabeau-Beale, Vincent J. Reid, Arica Hirsch, Catherine Hwang, James Pellicane, Robert Maganini, Sadia Khan, Dhara M. MacDermed, William Small, Karuna Mittal, Patrick Borgen, Charles Cox, Steven C. Shivers, Troy Bremer

**Affiliations:** 1 Department of Radiation Oncology, Taussig Cancer Institute, Cleveland Clinic, Cleveland, OH USA; 2 Nashville Breast Center, Nashville, TN USA; 3 PreludeDX, Laguna Hills, CA USA; 4 Michigan Healthcare Professionals, Farmington Hills, MI USA; 5 Center for Global Health, University of Toronto, Toronto, ON Canada; 6 Department of Radiation Oncology, Laura and Isaac Perlmutter Cancer Center, New York, NY USA; 7 Department of Radiation Oncology, James Cancer Center, Ohio State University, Columbus, OH USA; 8 Department of Surgery, Dana-Farber Cancer Institute, Boston, MA USA; 9 Schar Cancer Institute, Inova, Fairfax, VA USA; 10 Department of Radiation Oncology, University of Colorado School of Medicine, Aurora, CO USA; 11 Comprehensive Breast Care, Michigan Healthcare Professionals, Troy, MI USA; 12 Arizona Center for Cancer Care, Phoenix, AZ USA; 13 Department of Radiation Oncology, University of Pittsburgh, Pittsburgh, PA USA; 14 GenesisCare, Fort Myers, FL USA; 15 Mercy Medical Center, Baltimore, MD USA; 16 Department of Surgery, Houston Methodist, Sugar Land, TX USA; 17 Wellstar Radiation Oncology, Hiram, GA USA; 18 BayCare Medical Group, Tampa, FL USA; 19 St. Luke’s Hospital, Allentown, PA USA; 20 Northwell Health, New Hyde Park, NY USA; 21 Radiation Oncology Associates, Fairfax, VA USA; 22 Hall-Perrine Cancer Center, Cedar Rapids, IA USA; 23 Advocate Health Care, Park Ridge, IL USA; 24 AdventHealth, Orlando, FL USA; 25 Bon Secours, Richmond, VA USA; 26 AMITA Health, Bartlett, IL USA; 27 Hoag Breast Center, Irvine, CA USA; 28 Sutter Health, San Mateo, CA USA; 29 Department of Radiation Oncology, Loyola University, Chicago, IL USA; 30 PreludeDx, Laguna Hills, CA USA; 31 Maimonides Medical Center, Brooklyn, NY USA; 32 University of South Florida Morsani College of Medicine, Tampa, FL USA

**Keywords:** Ductal carcinoma in situ, Radiation therapy, Decision-making tools

## Abstract

**Background:**

Breast-conserving surgery (BCS) followed by adjuvant radiotherapy (RT) is a standard treatment for ductal carcinoma in situ (DCIS). A low-risk patient subset that does not benefit from RT has not yet been clearly identified. The DCISionRT test provides a clinically validated decision score (DS), which is prognostic of 10-year in-breast recurrence rates (invasive and non-invasive) and is also predictive of RT benefit. This analysis presents final outcomes from the PREDICT prospective registry trial aiming to determine how often the DCISionRT test changes radiation treatment recommendations.

**Methods:**

Overall, 2496 patients were enrolled from February 2018 to January 2022 at 63 academic and community practice sites and received DCISionRT as part of their care plan. Treating physicians reported their treatment recommendations pre- and post-test as well as the patient’s preference. The primary endpoint was to identify the percentage of patients where testing led to a change in RT recommendation. The impact of the test on RT treatment recommendation was physician specialty, treatment settings, individual clinical/pathological features and RTOG 9804 like criteria. Multivariate logisitc regression analysis was used to estimate the odds ratio (ORs) for factors associated with the post-test RT recommendations.

**Results:**

RT recommendation changed 38% of women, resulting in a 20% decrease in the overall recommendation of RT (*p* < 0.001). Of those women initially recommended no RT (*n* = 583), 31% were recommended RT post-test. The recommendation for RT post-test increased with increasing DS, from 29% to 66% to 91% for DS <2, DS 2–4, and DS >4, respectively. On multivariable analysis, DS had the strongest influence on final RT recommendation (odds ratio 22.2, 95% confidence interval 16.3–30.7), which was eightfold greater than clinicopathologic features. Furthermore, there was an overall change in the recommendation to receive RT in 42% of those patients meeting RTOG 9804-like low-risk criteria.

**Conclusions:**

The test results provided information that changes treatment recommendations both for and against RT use in large population of women with DCIS treated in a variety of clinical settings. Overall, clinicians changed their recommendations to include or omit RT for 38% of women based on the test results. Based on published clinical validations and the results from current study, DCISionRT may aid in preventing the over- and undertreatment of clinicopathological ‘low-risk’ and ‘high-risk’ DCIS patients.

**Trial Registration:**

ClinicalTrials.gov identifier: NCT03448926 (https://clinicaltrials.gov/study/NCT03448926).

**Supplementary Information:**

The online version contains supplementary material available at 10.1245/s10434-024-15566-5.

Ductal carcinoma in situ (DCIS) is a non-invasive malignancy of the breast diagnosed annually in about 60,000 women in the United States (US).^1–3^ In general, the majority of DCIS cases are screen-detected in the US and have an excellent long-term breast cancer-specific survival of around 97%.^4–7^ Most patients (about 40,000 annually) are treated with breast-conserving surgery (BCS) with or without radiotherapy (RT), with an in-breast recurrence (IBR) rate at 10 years of 10–20% with BCS alone, with half of the IBRs being invasive recurrences.^5,8,9^ RT reduces the IBR rate as part of breast-conserving therapy, but does not impact overall survival.^7,10^ Consequently, the treatment goal for DCIS is the prevention of subsequent breast cancer recurrences while taking into consideration patient preferences and quality of life.^11^

Given the non-invasive nature of DCIS, the lack of survival benefit with radiation, and the relatively low risk of recurrence for most cases of screen-detected DCIS, a key goal in the management of DCIS is to avoid overtreatment of patients who are not destined to recur after BCS without RT. For many, the absolute reduction of IBR may not be large enough to justify the risks, costs, and time associated with RT.^12,13^ However, omitting RT has also been associated with an elevated risk of IBR (including invasive breast cancer), raising concerns about undertreating patients.^7^ Unfortunately, current predictive models are insufficient in this respect as prospective studies using clinicopathologic factors alone have not clearly identified a ‘low-risk’ DCIS cohort who do not clinically benefit from RT, and low-risk DCIS patients still showed a 70–80% reduction in IBR risk with RT.^4,11–14,21^

To assess DCIS recurrence risk and to predict the benefit of the treatment, it is imperative to identify and validate methods that combine clinical/pathological features with new molecular factors.^15^ The reproducibility, predictive ability, and clinical utility of such a test are considerations necessary to support its clinical use.^16^

The DCISionRT test (7-gene biosignature) generates a continuous risk score (Decision score [DS]) on a 10-point scale and provides 10-year IBR risks after BCS with and without RT.^17^ The test demonstrated high analytical sensitivity, specificity, accuracy, reproducibility, and precision,^18^ and was prognostic of IBR risk and predictive of RT benefit in multiple validation studies.^17,19–22^ These clinical validation studies, which include a large randomized clinical trial (SweDCIS), demonstrate that patients who were classified by DCISionRT as being at low risk received no significant benefit from RT, whereas patients with higher DS results had much higher IBR rates, which were significantly reduced with adjuvant RT. The test had a 99% negative predictive value for RT benefit for patients in the low-risk group, while 96% of all patients benefiting from RT were correctly classified into the high-risk group.^22^

In the PREDICT study, we evaluated the clinical utility of the DCISionRT test in routine clinical practice by comparing the pre- and post-test clinician recommendations for RT in more than 2000 DCIS patients considering breast conservation. The impact of patient preference, clinical factors, race/ethnicity, pathologic features, and low-risk clinicopathologic criteria on the physician’s treatment recommendation are also assessed. Interim results from the study were published in 2021,^20^ with 539 patients evaluated. Herein, we present results, after the enrollment of patients was complete from the full cohort of more than 2000 patients, to evaluate the change in the radiation recommendation, including stratification based on patients with ‘low-risk’ and ‘high-risk’ clinicopathology features.

## Methods and Materials

The PREDICT study is an observational, prospective, multicenter study of women diagnosed with DCIS. Patients were enrolled from 63 US academic centers and community practices. The primary goal of the study was to assess the clinical utility of the DCISionRT test through its impact on physician decision making for RT treatment recommendations overall and compared with traditional clinicopathologic factors. DCISionRT is a commercial Multianalyte Assay with Algorithmic Analyses (MAAA) by PreludeDx (Laguna Hills, CA) that is performed for women diagnosed with breast ductal carcinoma in situ (DCIS), using a formalin-fixed, paraffin-embedded DCIS tissue specimen. Interim results from the study were published in 2021^20^ with 539 patients evaluated. Herein, we present the results after the enrollment of patients was complete. Eligible patients included women ≥25 years of age diagnosed with DCIS who were considering breast-conserving treatment. Exclusion criteria included previous breast malignancy, evidence of invasive breast cancer (including microinvasion or lymph node involvement), Paget’s disease, pregnancy, serious comorbidities, and insufficient tumor tissue to perform testing. Patients with positive margins or patients for whom the pre- and post-test treatment recommendations were reported by different clinician specialties were excluded from the analysis. The study was approved by the WCG Institutional Review Board (WCG IRB) and by Institutional Review Boards of the 63 participating centers. All participants signed informed consent and the study protocol was registered in the ClinicalTrials.gov database (NCT03448926).

Sample processing and analytical and clinical test validations have been previously described.^17–22^ For all patients, demographic information and clinicopathologic characteristics were recorded. RT treatment recommendation was made by clinicians (yes or no RT) prior to the clinician receiving the DCISionRT test result (pre-test recommendation). For each patient, one paired pre-test/post-test treatment recommendation was made by a surgeon (independently), a radiation oncologist (independently), or with a tumor board. The paired pre- and post-test treatment recommendations were provided by a clinician in the same specialty (surgeon or radiation oncologist treating the patient). Patient preference for or against RT was recorded post-DCISionRT testing. No standardized criteria for recommending or not recommending RT were implemented.

The primary endpoint of the study was the frequency in the change in clinicians’ recommendations for RT from pre- to post-test. McNemar’s test for paired data was used to assess the significance of the changes. The impact of the DCISionRT results on clinician treatment recommendations was evaluated for various patient subsets defined by clinical factors and pathologic features (e.g., institution-determined tumor grade and size). Patients were also evaluated according to clinicopathologic criteria for ‘low risk’, including RTOG 9804-like criteria (low or intermediate grade, size ≤2.5 cm, no positive margins [ink on tumor], non-palpable, screen-detected) and ECOG E5194-like criteria (low or intermediate grade, size ≤2.5 cm, no positive margins).^23,24^ Summary data for treatment recommendations were also grouped by physician specialty: surgeons (independently), versus radiation oncologists (independently), versus radiation oncologists (independently) or tumor boards.

Multivariate logistic regression analysis was used to estimate the odds ratio (OR) for factors associated with the post-test RT recommendations, including age (≥70 years vs. <50 years vs. 50–69 years), and local pathology assessment of grade (high vs. low or intermediate grade), tumor size (>1 to ≤2.5 cm vs. >2.5 cm vs. ≤1 cm), detection (clinical vs. mammographic screening), ethnicity (Hispanic vs. non-Hispanic), race (African American, Caucasian, other), and patient preference (post-test), as well as clinician specialty (surgeon vs. radiation oncologist) and clinical setting (regional hospital vs. independent practice vs. academic). Post-test covariates also included the DS, assessed on both a continuous and categorical basis. Summary data analyses included Chi-square, Fisher’s exact test, and t-test statistics. All analyses were performed using R.^25^ Statistical significance was assessed using the McNemar’s or Chi-square tests, Fisher’s exact test, and the t-test where appropriate.

## Results

Overall, 2007 eligible patients of the 2496 enrolled from 63 centers between February 2018 and January 2022 formed our study cohort (Online Resource Fig. 1, Online Resource Table 1). Patient and tumor characteristics are presented in Table 1. The median age at diagnosis was 62 years (interquartile range [IQR] 53–70 years). Median extent of DCIS was 0.7 cm (IQR 0.4–1.4 cm), 32% of cases were high nuclear grade, and 7% were hormone receptor-negative. Fifty-eight percent of patients met the RTOG 9804-like criteria for ‘low risk’.

**Fig. 1:**
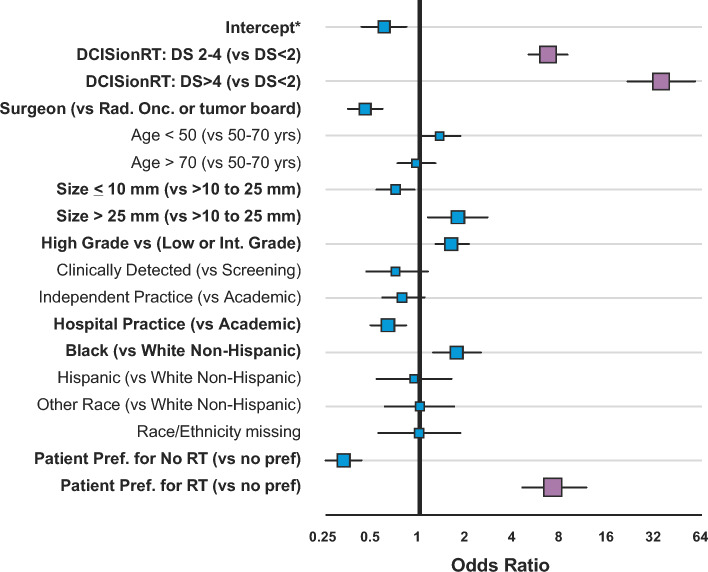
Factors associated with RT recommendations post-DCISionRT testing. * Intercept (reference group): DS 0–2, age 50–69 years, low or intermediate grade, size 11–25 mm, screening detected, radiation oncologist (independent) or tumor board at an academic center, Caucasian non-Hispanic patient with no patient preference for RT (see MVA Online Resource Table 2). *RT* radiotherapy, *DS* decision score, *Rad. Onc.* radiation oncologist, *Int.* intermediate, *pref* preference

**Table 1:** Clinical and pathologic characteristics stratified by clinician specialty

	All^a^		RadOnc (independent)		Surgeon (independent)^b^		RadOnc (independent) or Tumor Board^b^		RadOnc vs. surgeon
Clinpath factor	*N, *%		*N, *%		*N, *%		*N, *%		*p*-Value^b^
All cases	2007		935		738		1263		
Age, years									
<50	337	17	155	17	122	17	215	17	0.28
50–69	1148	57	539	57	439	59	708	56	
≥70	522	26	241	26	177	24	340	27	
Nuclear grade									
Low	339	17	162	17	120	16	217	17	0.81
Intermediate	1020	51	472	50	382	52	637	50	
High	648	32	301	33	236	32	409	33	
Tumor size, cm									
≤1	1336	67	624	67	497	67	834	66	0.60
1–2.5	478	23	233	25	167	23	310	25	
>2.5	193	10	78	8	74	10	119	9	
Tumor necrosis^c^									
Present	1076	54	501	54	414	56	659	52	0.89
Absent	446	22	198	21	169	23	275	22	
RTOG 9804^c^									
‘Good risk’^d^	1163	58	540	58	438	59	722	57	
Not ‘good risk’	832	41	385	41	299	41	530	42	0.47
Missing	12	1	10	1	1	0	11	1	
ECOG E5194									
Low or intermediate grade	1199	60	562	60	445	60	751	59	0.92
High grade	362	18	176	19	130	18	230	18	
Neither	446	22	197	21	163	22	282	22	

^a^Includes patients with treatment recommendations reported by radiation oncologists, surgeons, and medical oncologists (*n* = 6)

^b^Compares patients with treatment recommendations reported by surgeons (independent) versus (radiation oncologists [independent] or tumor board)

^c^Missing data

^d^RTOG 9804-like ‘good risk’ criteria are defined as low or intermediate grade, size ≤2.5 cm, negative margins (no ink on tumor), non-palpable, screen-detected

*RadOnc* radiation oncologist

Pre-test, 71% of patients (*n* = 1424/2007) were initially recommended to receive adjuvant RT compared with 51% of patients (*n* = 1024/2007) who were recommended to receive RT post-test (Table 2), a net reduction of 20% (*p* < 0.001). Overall, RT recommendations were changed for 38% of patients (*n* = 765/2007) [*p* < 0.001]. Of 1424 patients recommended to receive RT pre-test, 584 (41%) had a change in recommendation to not receive RT post-test. Of the 583 patients recommended to not receive RT pre-test, 31% (*n* = 181/583) were recommended to receive RT post-test. As previously shown,^20^ the percentage of patients recommended RT post-test varied with DS, and was 33% for DS <3 and 85% for DS ≥3 (Table 2). Similar results were observed when the impact of the continuum of DS results on treatment recommendations was evaluated, wherein the percentage of patients recommended RT post-test was 29% for DS <2, 66% for DS 2–4, and 91% for DS >4 (Table 3). On multivariable analysis for recommending RT using DS <2 as the reference group, the ORs for RT recommendation were 6.8 (95% confidence interval [CI] 5.2–8.9) for DS 2–4 and 36.4 (95% CI 22.7–60.9) for DS >4 (Fig. 1, Online Resource Table 2).^20^

**Table 2:** Radiation therapy recommendations pre- and post-DCISionRT testing stratified by dichotomous DCISionRT risk groups

DS range	*n*	RT recommended			Pre- to post-test change in RT recommended		Total change in RT recommended		
		Pre-test (%)	Post-test (%)	Net change (%)	Yes to no (%)	No to yes (%)	Overall change (%)	95% CI	*p*-Value
All^a^	2007	71	51	−20	41	31	38	36–40%	< 0.0001
DS ≤3	1307	73	33	−40	58	8	45	42–47%	< 0.0001
DS >3	700	68	85	17	7	67	26	23–26%	< 0.0001

^a^Includes patients with treatment recommendations reported by surgeons or oncologists

*RT* radiotherapy, *DS* decision score, *CI* confidence interval

**Table 3:** Radiation therapy recommendations pre- and post-DCISionRT testing stratified by DS range

DS range	*n*	RT recommended			Pre- to post-test change in RT recommended		Total change in RT recommended		
		Pre-test (%)	Post-test (%)	Net change (%)	Yes to no (%)	No to yes (%)	Overall change (%)	95% CI	*p*-Value
All^a^	2007	71	51	−20	41	31	38	36–40%	< 0.0001
DS <2	1026	72	29	−43	63	7	47	44–50%	< 0.0001
DS 2–4	703	66	66	0	24	49	33	29–36%	0.84
DS >4	278	80	91	11	4	75	18	14–23%	< 0.0001

^a^Includes patients with treatment recommendations reported by surgeons or oncologists

*DS* decision score, *RT* radiotherapy, *CI* confidence interval

On multivariable analysis, the DCISionRT test was the strongest predictor of RT recommendation post-test (OR 22.2 for DS >3 vs. DS ≤3, 95% CI 16.3–30.7). Patient preference for RT (vs. no preference) was the second strongest predictor (OR 7.8, 95% CI 4.9–12.7) (see Table 4). After discussing the test result with their physician, 22% of patients expressed a preference not to receive RT and 10% of patients expressed a preference to receive RT. The influence of patient preference on the likelihood of RT recommendation was greatest for those with lower DS results (DS <2) (see Online Resource Fig. 2).

**Table 4:** Multivariable logistic regression analysis of dichotomous DCISionRT risk groups, clinicopathologic factors, race/ethnicity, and other factors influencing the likelihood of RT treatment recommendations

	OR	Lower CI	Upper CI
Intercept^a^	0.8	0.6	1.1
DCISionRT (DS >3 vs. DS ≤3)	**22.2**	**16.3**	**30.7**
Surgeon (vs. radiation oncologist)	**0.5**	**0.4**	**0.6**
Age <50 years (vs. age 50–69 years)	1.1	0.8	1.6
Age ≥70 years (vs. age 50–69 years)	**0.6**	**0.5**	**0.9**
Size ≤10 mm (vs. >10 to 25 mm)	**0.6**	**0.5**	**0.8**
Size >25 mm (vs. >10 to 25 mm)	**1.8**	**1.2**	**2.8**
High grade (vs. low or intermediate grade)	**2.6**	**2.0**	**3.3**
Clinical detection (vs. mammographic detection)	1.0	0.6	1.5
Independent practice (vs. academic center)	**0.6**	**0.4**	**0.9**
Regional hospital practice (vs. academic center)	**0.6**	**0.4**	**0.8**
Black (vs. Caucasian non-Hispanic)	**1.8**	**1.2**	**2.6**
Hispanic (vs. Caucasian non-Hispanic)	0.8	0.5	1.5
Other non-Caucasian race (vs. Caucasian non-Hispanic)	1.0	0.6	1.7
Race missing (vs. Caucasian non-Hispanic)	1.1	0.6	2.0
Patient preference for no RT (vs. no patient preference)	**0.3**	**0.2**	**0.4**
Patient preference for RT (vs. no patient preference)	7.8	4.9	12.7

^a^Intercept (reference group): DS ≤3, age 50–69 years, low or intermediate grade, size 11–25 mm, screening detected, radiation oncologist (independent) or tumor board at an academic center, Caucasian non-Hispanic patient with no patient preference for RT

*OR* odds ratio, *CI* confidence interval, *DS* decision score, *RT* radiotherapy

On multivariable analysis, surgeons were less likely to recommend RT than radiation oncologists (OR 0.5, 95% CI 0.4–0.6) (Table 4), as illustrated in Online Resource Fig. 2. Surgeons changed their RT recommendation from yes (pre-test) to no (post-test) more frequently than radiation oncologists (55% vs. 33%) (Online Resource Table 3). Multivariable analysis also indicated that clinicians at regional hospitals or in independent practices were somewhat less likely to recommend RT than clinicians at academic centers (OR 0.6) (Table 4). The 7-gene predictive biosignature (OR 22.2) had an eightfold greater influence than standard clinicopathologic risk factors on RT recommendation post-test (Table 4). Specifically, high tumor grade and Black race increased the chance for being recommended RT post-test, with ORs of 2.6 (95% CI 2.0–3.3) and 1.8 (95% CI 1.2–2.6), respectively. Similar results were observed when the multivariable logistic regression analysis of continuous DSs and clinicopathologic factors was performed for the likelihood of RT recommendation (Online Resource Table 4).

The DCISionRT test significantly influenced treatment recommendations post-test among patients with standard ‘low-risk’ or ‘high-risk’ clinicopathologic factors (27–45%) (see Table 5). Young age and high tumor grade were associated with high recommendation rates for RT pre-test (80% and 87%, respectively) and were also associated with greater net reductions in the recommendation for RT post-test (reductions of 35% and 25%, respectively). Tumor size (> 2.5 cm) was also associated with high pre-test recommendation rates for RT (90%), but this declined to 70% post-test. For older patients (age >70 years), recommendation rates for RT were lower pre-test than for women aged 50–69 years, and there was a small net difference in the RT recommendation rate post-test; however, clinicians changed their recommendation to include or omit RT for 35% of these women.

**Table 5:** Impact of DCISionRT on treatment recommendation stratified by clinicopathologic criteria

Clinicopathologic criteria	*n*	RT recommended			Pre- to post-test change in RT recommended		Total change in RT recommended		
		Pre-test (%)	Post-test (%)	Net change (%)	Yes to no (%)	No to yes (%)	Overall change (%)	95% CI	*p*-Value
All^a^	2007	71	51	−20	41	31	38	36–40%	< 0.0001
Age, years									
< 50	337	80	45	−35	46	7	38	33–43%	< 0.0001
50–69	1148	74	47	−27	45	23	39	37–42%	< 0.0001
≥ 70	522	59	64	5	25	49	35	31–39%	0.054
Nuclear grade									
Low	339	54	45	−9	49	39	45	39–50%	0.018
Intermediate	1020	67	45	−21	46	27	40	37–43%	< 0.0001
High	648	87	62	−25	33	29	32	29–36%	< 0.0001
Tumor size, cm									
≤ 1	1336	65	45	−20	46	28	40	37–42%	< 0.0001
1–2.5	478	81	60	−21	36	44	38	34–42%	< 0.0001
> 2.5	193	90	69	−21	26	32	27	21–33%	< 0.0001
RTOG 9804-like^b^									
‘Good risk’^c^	1163	62	43	−19	49	31	42	40–45%	< 0.0001
Not ‘good risk’	832	84	61	−23	33	30	32	29–36%	< 0.0001
ECOG E5194-like^d^									
Low or intermediate grade	1199	62	43	−19	49	31	42	39–45%	< 0.0001
High grade	362	83	55	−28	38	22	36	31–41%	< 0.0001
Not good risk	446	88	69	−19	28	42	29	25–34%	< 0.0001
Alternative criteria									
Age ≥50 years, size ≤2.5 cm, ER-positive, no ink on tumor	1016	66	49	−17	44	35	41	38–44%	< 0.0001

^a^Includes patients with treatment recommendations reported by surgeons or oncologists

^b^Missing data for some patients

^c^RTOG 9804-like ‘good risk’ criteria are defined as low or intermediate grade, size ≤2.5 cm, negative margins (no ink on tumor), non-palpable, screen-detected

^d^ECOG 5195-like criteria are defined as low or intermediate grade, intermediate grade, size ≤2.5 cm, margin-negative (no ink on tumor), or high grade, size ≤1 cm, margin-negative (no ink on tumor)

*RT* radiotherapy, *CI* confidence interval, *ER* estrogen receptor

Prior to testing, 62% of patients who met the RTOG 9804-like criteria for ‘low risk’ DCIS were recommended to receive adjuvant RT (Table 5). Post-test, 43% of patients were recommended to receive RT (net reduction of 19%, *p* < 0.001). Thirty-one percent of RTOG 9804 ‘low-risk’ patients who were not recommended RT were recommended RT post-test. Similar findings were seen using the ECOG E5194-like ‘low-risk’ criteria (age ≥50 years, estrogen receptor-positive DCIS, size ≤ 2.5 cm, and negative margins). In contrast, for patients not meeting the RTOG 9804-like criteria, 84% were recommended RT pre-test and 61% were recommended RT post-test, where RT recommendations were changed for 32% of women post-test (*p* < 0.001). Similar results were observed for patients meeting high-grade ECOG E5194-like criteria. Interestingly, in each clinicopathological (CP) risk group, the recommendation of RT changed based on the classification of patients in the DS groups regardless of the CP criteria. More than 50% of patients with a DS <2 who were classified in the CP high- or low-risk criteria and who were recommended RT pre-test were not recommended RT post-test. In line with this, more than approximately 60% of patients with DS >4 in the CP high- or low-risk criteria who were not recommended RT pre-test were recommended RT post-test (Online Resource Table 5).

Radiation oncologists recommended RT with boost to a similar percentage of patients pre-test (24%) and post-test (19%); however, the recommendation for RT with boost was changed for 15% of patients post-test (Online Resource Table 6). The corresponding post-test recommendation for RT with boost increased with increasing DS results (*p* < 0.001), where the percentage of patients recommended RT with boost was 13% for DS <2, 20% for DS 2–4, and 41% for DS >4 (Online Resource Fig. 3).

## Discussion

The results of the present analysis of over 2000 patients with DCIS demonstrate several key findings. First, the use of the DCISionRT test to assess recurrence risk and the benefit of RT led to changes in treatment recommendations for 38% of all women, including a net reduction of 20% in the number of women recommended by their clinician to have RT. Second, 31% of patients who were originally not recommended to receive RT were recommended to receive RT post-test. Finally, when evaluating factors associated with RT recommendation post-test, the DCISionRT results had the greatest impact of all other factors. These findings, coupled with the previously published analytical and clinical validation studies of the DCISionRT test, support that DCISionRT testing should be considered to aid in shared treatment decision making for most DCIS patients considering BCS.^17,19–22^

Previous studies utilizing traditional clinical and pathologic features alone have failed to clearly and consistently identify a low-risk group of patients with DCIS who may safely forgo RT after BCS, i.e., a group of patients predicted to have no significant decline in the risk of recurrence with RT. This study included many patients considered to be low risk by current definitions: 58% met the RTOG 9804-like low-risk criteria and 60% met ECOG E5194-like low/intermediate-grade criteria.^23,24^ Results of the present analysis demonstrate the unique ability of the DCISionRT test to provide 10-year risk and RT benefit profiles that aided shared treatment decision making for patients with ‘low-risk’ clinicopathologic features. For example, there was an overall change in the recommendation to receive RT in 42% of those patients meeting RTOG 9804-like low-risk criteria, where 38% of these CP low-risk patients were initially not recommended to receive RT pre-test, but one-third of this group were recommended RT post-test. Similar findings were seen for patients who met the low/intermediate-grade ECOG-like low-risk criteria, as well as for patients aged 50 years or older and tumors 2.5 cm or less who were ER-positive and had no positive margins. Likewise, among patients with conventional ‘high risk’ clinicopathologic features, the DCISionRT test refined risk and treatment benefit profiles based on the molecular biosignature, which aided shared decision making. For example, 33% and 38% of patients not meeting RTOG 9804-like criteria or meeting E5194-like high-grade criteria, respectively, who were recommended RT pre-test were subsequently recommended no RT post-test.

In view of previously published data, the above findings highlight several important points. First, for patients with ‘low-risk’ clinicopathologic features, use of the DCISionRT test led to a substantial percentage of such patients being reclassified with significantly higher predicted recurrence risk. These data suggest that using clinicopathologic features alone may underestimate recurrence risk for many patients. On the other hand, among patients with ‘high-risk’ clinicopathologic features, the test identified that a substantial percentage of patients who had a low biosignature estimated risk and RT benefit profile and were subsequently recommended by their clinicians not to have RT. Combined with prior DCISionRT validations, this study supports that the DCISionRT test provides useful ‘unique’ information that frequently changes clinician treatment recommendations. As such, rather than a simple de-escalation of adjuvant RT, this study demonstrates that the predictive biosignature provides information frequently used to balance the benefit and risk of treatment for individual patients.

Decision making regarding the utilization of RT after BCS should be based on a shared decision-making model. Applying the DCISionRT test allows for a patient to receive personalized information regarding the optimal management of their DCIS, including the risk of recurrence and predictive benefit of RT. Studies have shown that different patients view risks and benefits differently, in that one patient may find a given recurrence risk without RT to be acceptable while another may not. This is consistent with the findings of this study, which demonstrated that patient preference for RT was the second most important factor apparent in the decision for RT. As such, as part of the shared decision-making process, clinicians should educate patients on the DCISionRT test and its outcomes while assessing patient preference for RT both prior to and following test results. In this study, patients who had reported a preference for RT were twice as likely to be recommended RT as those with no preference. Again, this demonstrates the need for clinicians to ascertain their patient’s risk tolerance and individual preference for RT treatment given their risks and benefits, consistent with National Comprehensive Cancer Network (NCCN) guidelines emphasizing shared decision making.

Finally, the tailoring of treatment for DCIS patients is not simply a binary decision of radiation versus no radiation. In the present study, about 20% of patients were recommended RT with boost by their radiation oncologist pre- and post-test; however, the boost recommendation was changed for 15% of patients’ post-test. Recommendation for boost varied with DS, ranging from 13% of patients with DS <2 to 41% of patients with DS >4. While the DCISionRT test has not been specifically validated for the benefit of RT with boost versus no boost, the findings of this study suggest the potential for DCISionRT to further guide RT decision making beyond a simple yes or no for adjuvant RT based on the reported risk profile. Studies are planned to evaluate the interaction of DCISionRT and dose escalation on IBR rates.

There are limitations to the present analysis. The study endpoint was not designed to evaluate clinical outcomes following DCISionRT testing but rather the impact of the test result on treatment recommendation. As such, IBR rates for patients in the study are not yet available; however, previous studies provided actual 10-year IBR rates for patients based on DCISionRT testing.^17,19,21,22^ Additionally, the study primarily focused on patients in the US and may not fully represent global clinical practice. Finally, as the study focused on patient/provider recommendations, decision making was not randomized or strictly rules-based but rather based on shared decision making with the patient. Future studies will evaluate the actual recurrence risk rates in patients who did and did not receive RT after discussing the DCISionRT test result with their physician. It will also be important to assess whether the DCISionRT test results influence the patient’s decision to have a mastectomy rather than BCS.

## Conclusions

The test provided information that changed treatment recommendations both for and against RT use in a large population of women with DCIS treated in a variety of clinical settings. Overall, clinicians’ changed their recommendations to include or omit RT for 38% of women based on the test results. Based on published clinical validations and the results from the current study, DCISionRT may aid in preventing the over- and undertreatment of CP low- and high-risk DCIS patients.

### Supplementary Information

Below is the link to the electronic supplementary material.

Supplementary file1 (DOCX 579 kb)
