# Supplementary material for: The Clinical Utility of a 7-Gene Biosignature on Radiation Therapy Decision Making in Patients with Ductal Carcinoma In Situ Following Breast-Conserving Surgery: An Updated Analysis of the DCISionRT® PREDICT Study
Source: Ann Surg Oncol. 2024 Jun 25;31(9):5919–28. doi: 10.1245/s10434-024-15566-5 (PMC11300542; doi:10.1245/s10434-024-15566-5)

**Supplemental Tables and Figures:**

[**Supplemental Table 1:** Clinicopathologic features stratified by patients inclusion vs. exclusion from analysis 2](#_Toc144293073)

[**Supplemental Table 2:** Multivariable Logistic Regression Analysis of DCISionRT test ranges and clinicopathologic factors for the likelihood of RT treatment recommendation 3](#_Toc144293074)

[**Supplemental Table 3:** Impact of DCISionRT on Adjuvant Radiation Recommendations Stratified by Clinicopathologic Features 4](#_Toc144293075)

[**Supplemental Table 4:** Multivariable Logistic Regression Analysis of Continuous Decision Scores (DS) and Clinicopathologic factors for the likelihood of RT treatment
recommendation 5](#_Toc144293076)

[**Supplemental Table 5:** Change in RT treatment recommendation after DCISionRT testing by DS ranges and Clinician Specialty 6](#_Toc144293077)

[**Supplemental Table 6:** Change in RT boost recommendation by Radiation Oncologists after DCISionRT testing by DS ranges 7](#_Toc144293078)

**Supplemental Table 7:** Impact of DCISionRT on Adjuvant Radiation Recommendations Stratified by Clinicopathologic Groups and DS Scores.............................................................

[**Supplemental Figure 1:** REMARK Diagram 8](#_Toc144293082)

[**Supplemental Figure 2:** Influence of continuous DS and patient preference on likelihood of recommendations for RT post-test reported by clinician specialty 9](#_Toc144293083)

[**Supplemental Figure 3:** Percentage of patients recommended RT with and without boost by DS ranges by radiation oncologists (independently) or tumor board 10](#_Toc144293084)

**Supplemental Table 1:** Clinicopathologic features stratified by patients inclusion vs. exclusion from analysis.

| **Clinpath  Factor** | **All Cases with Complete Data** | | **Cases  Excluded*** | | **Cases  Analyzed** | | **p-Value** |
| --- | --- | --- | --- | --- | --- | --- | --- |
|  | **n** | **%** | **n** | **%** | **n** | **%** |  |
| **All Cases** | **2305** |  | **298** |  | **2007** |  |  |
| **Age** |  |  |  |  |  |  |  |
| Under 50 | 368 | 16% | 31 | 10% | 337 | 17% | 0.018 |
| 50 - 69 | 1334 | 58% | 186 | 63% | 1148 | 57% |  |
| 70 and over | 603 | 26% | 81 | 27% | 522 | 26% |  |
| **Median Age** | 62 |  | 63 |  | 62 |  | 0.027 |
| **Nuclear Grade** |  |  |  |  |  |  |  |
| Low Grade | 386 | 17% | 47 | 15% | 339 | 17% | 0.52 |
| Intermediate Grade | 1165 | 51% | 145 | 49% | 1020 | 51% |  |
| High Grade | 754 | 33% | 106 | 36% | 648 | 32% |  |
| **Tumor Size** |  |  |  |  |  |  |  |
| ≤ 1 cm | 1515 | 66% | 179 | 60% | 1336 | 67% | 0.040 |
| 1 - 2.5 cm | 569 | 25% | 91 | 31% | 478 | 24% |  |
| > 2.5 cm | 221 | 10% | 28 | 9% | 193 | 10% |  |
| **Median Size (mm)** | 7 |  | 9 |  | 7 |  | 0.085 |
| **Margin Status** |  |  |  |  |  |  |  |
| Positive | 51 | 2% | 51 | 17% | 0 | 0% | <0.001 |
| Negative | 2254 | 98% | 247 | 83% | 2007 | 100% |  |
| **Tumor Necrosis ^#^** |  |  |  |  |  |  |  |
| Present | 1231 | 53% | 155 | 52% | 1076 | 54% | 0.68 |
| Absent | 519 | 23% | 73 | 24% | 446 | 22% |  |
| Missing | 555 | 24% | 70 | 23% | 485 | 24% |  |
| **RTOG 9804-like ^#^** |  |  |  |  |  |  |  |
| “Good risk” | 1301 | 56% | 138 | 46% | 1163 | 58% | <0.001 |
| Not “good risk” | 986 | 43% | 160 | 54% | 832 | 41% |  |
| Missing | 18 | 1% | 0 | 0% | 12 | 1% |  |
| **ECOG E5194-like** |  |  |  |  |  |  |  |
| Low or Intermediate Grade | 1340 | 58% | 141 | 47% | 1199 | 60% | <0.001 |
| High Grade | 405 | 18% | 43 | 14% | 362 | 18% |  |
| Not Good Risk | 560 | 24% | 114 | 38% | 446 | 22% |  |
| **Race** |  |  |  |  |  |  |  |
| White | 1858 | 81% | 227 | 77% | 1631 | 82% | 0.02 |
| Black | 265 | 11% | 40 | 13% | 225 | 11% |  |
| Asian | 128 | 6% | 26 | 9% | 102 | 5% |  |
| Other | 8 | 0% | 1 | 0% | 7 | 0% |  |
| Missing | 46 | 2% | 4 | 1% | 42 | 2% |  |

* Patients with positive margins or non-paired treatment recommendation (pre-test/post-test CRFs completed by different clinical specialty).

# Missing data for some patients.

**Supplemental Table 2:** Multivariable Logistic Regression Analysis of DCISionRT test ranges and clinicopathologic factors for the likelihood of RT treatment recommendation

|  | **OR** | **Lower CI** | **Upper CI** |
| --- | --- | --- | --- |
| **Intercept *** | **0.6** | **0.4** | **0.8** |
| DS 2-4 (vs. DS <2) | **6.8** | **5.2** | **8.9** |
| DS >4 (vs. DS <2) | **36.4** | **22.7** | **60.9** |
| Surgeon (vs. Radiation Oncologist) | **0.5** | **0.4** | **0.6** |
| **Age <50 yrs (vs. Age 50-69)** | **1.4** | **1.0** | **1.9** |
| **Age ≥70 yrs (vs. Age 50-69)** | **1.0** | **0.7** | **1.3** |
| **Size ≤10 mm (vs. >10 to 25 mm)** | **0.7** | **0.6** | **0.9** |
| **Size >25 mm (vs. >10 to 25 mm)** | **1.8** | **1.2** | **2.8** |
| **High Grade (vs. Low or Intermediate Grade)** | **1.6** | **1.3** | **2.1** |
| Clinical Detection (vs. Mammographic Detection) | 0.7 | 0.5 | 1.1 |
| Independent Practice (vs Academic Center) | 0.8 | 0.6 | 1.1 |
| Hospital Practice (vs Academic Center) | **0.6** | **0.5** | **0.8** |
| Black (vs. Caucasian non-Hispanic) | **1.8** | **1.3** | **2.6** |
| Hispanic (vs. Caucasian non-Hispanic) | 0.9 | 0.5 | 1.7 |
| Other Non-Caucasian Race (vs. Caucasian non-Hispanic) | 1.0 | 0.6 | 1.7 |
| Race Missing (vs. Caucasian non-Hispanic) | 1.0 | 0.6 | 1.9 |
| **Patient Preference for No RT (vs. no patient preference)** | **0.3** | **0.2** | **0.4** |
| **Patient Preference for RT (vs. no patient preference)** | **7.4** | **4.7** | **12.1** |

* Intercept (reference group): DS 0-2, age 50-69, low or intermediate grade, Size 11 to 25 mm, screening detected, radiation oncologist (independent) or tumor board at an academic center, Caucasian non-Hispanic patient with no patient preference for RT.

**(see forest plot** [Figure 1](#_Supplemental_Figure_1)**)**

**Supplemental Table 4:** Multivariable Logistic Regression Analysis of Continuous Decision Scores (DS) and Clinicopathologic factors for the likelihood of RT treatment recommendation

|  | **OR** | **Lower CI** | **Upper CI** |
| --- | --- | --- | --- |
| **Intercept*** | 0.3 | 0.2 | 0.4 |
| **DS as a continuous variable (0.8 - 10)** | **2.3/unit** | **2.1/unit** | **2.6/unit** |
| **Surgeon (vs. Radiation Oncologist)** | **0.5** | **0.4** | **0.6** |
| **Age <50 yrs (vs. Age 50-69)** | **1.4** | **1.00** | **1.9** |
| Age ≥70 yrs (vs. Age 50-69) | 0.8 | 0.6 | 1.1 |
| **Size ≤10 mm (vs. >10 to 25 mm)** | **0.7** | **0.6** | **0.9** |
| **Size >25 mm (vs. >10 to 25 mm)** | **1.7** | **1.1** | **2.7** |
| **High Grade (vs. Low or Intermediate Grade)** | **1.7** | **1.4** | **2.2** |
| Clinical Detection (vs. Mammographic Detection) | 0.8 | 0.5 | 1.2 |
| Independent Practice (vs Academic Center) | 0.8 | 0.6 | 1.1 |
| **Hospital Practice (vs Academic Center)** | **0.6** | **0.5** | **0.8** |
| **Black (vs. Caucasian non-Hispanic)** | **1.8** | **1.3** | **2.6** |
| Hispanic (vs. Caucasian non-Hispanic) | 1.0 | 0.5 | 1.7 |
| Other Non-Caucasian Race (vs. Caucasian non-Hispanic) | 1.0 | 0.6 | 1.6 |
| Race Missing (vs. Caucasian non-Hispanic) | 1.1 | 0.6 | 2.0 |
| **Patient Preference for No RT (vs. no patient preference)** | **0.3** | **0.2** | **0.4** |
| **Patient Preference for RT (vs. no patient preference)** | **7.7** | **4.9** | **12.7** |

Intercept (Reference group): Age 50-69, low or intermediate grade, Size 11 to 25 mm, screening detected, radiation oncologist (independent) or tumor board at an academic center, Caucasian non-Hispanic patient with no patient preference for RT.

**Supplemental Table 3** Change in RT treatment recommendation after DCISionRT testing by DS ranges and Clinician Specialty

|  |  | **RT Recommended** | | | **Pre- to Post-Test Change in RT Recommended** | | **Total  Change in RT Recommended** | | |
| --- | --- | --- | --- | --- | --- | --- | --- | --- | --- |
| **Recommending Clinician** | **n** | **Pre-Test (%)** | **Post-Test (%)** | **Net  Change (%)** | **Yes to No (%)** | **No to Yes (%)** | **Overall  Change (%)** | **95% CI** | **p-Value** |
| **All *** | 2007 | 71% | 51% | -20% | 41% | 31% | 38% | 36-40% | <.0001 |
| Surgeons (independently) | 738 | 71% | 40% | -31% | 55% | 28% | 47% | 44-51% | <.0001 |
| Radiation Oncologists (independently) | 935 | 73% | 55% | -18% | 36% | 31% | 35% | 31-37% | <.0001 |
| Radiation Oncologists (independently) or  Tumor Board | 1263 | 71% | 57% | -14% | 33% | 33% | 33% | 30-35% | <.0001 |
| **DS <2 *** | **1026** | **72%** | **29%** | **-43%** | **63%** | **7%** | **47%** | **44-50%** | **<.0001** |
| Surgeons (independently) | 370 | 74% | 16% | -58% | 80% | 2% | 59% | 54-64% | <.0001 |
| Radiation Oncologists (independently) | 485 | 74% | 33% | -41% | 57% | 5% | 43% | 39-48% | <.0001 |
| Radiation Oncologists (independently) or  Tumor Board | 654 | 71% | 37% | -35% | 53% | 10% | 40% | 37-44% | <.0001 |
| **DS 2-4 *** | **703** | **66%** | **66%** | **0%** | **24%** | **49%** | **33%** | **29-36%** | **0.8989** |
| Surgeons (independently) | 264 | 65% | 55% | -10% | 39% | 43% | 40% | 34-46% | 0.012 |
| Radiation Oncologists (independently) | 325 | 68% | 73% | 6% | 17% | 52% | 28% | 24-33% | 0.0061 |
| Radiation Oncologists (independently) or  Tumor Board | 436 | 67% | 74% | 7% | 15% | 52% | 28% | 24-32% | 0.0062 |
| **DS >4 *** | **278** | **80%** | **91%** | **11%** | **4%** | **75%** | **19%** | **14-23%** | **<.0001** |
| Surgeons (independently) | 106 | 77% | 89% | 11% | 7% | 75% | 23% | 16-31% | 0.0143 |
| Radiation Oncologists (independently) | 125 | 83% | 94% | 11% | 2% | 76% | 14% | 9-21% | 0.0010 |
| Radiation Oncologists (independently) or  Tumor Board | 173 | 82% | 93% | 11% | 3% | 75% | 16% | 11-22% | 0.0002 |

* Includes patients with treatment recommendations by radiation oncologists, surgeons, and medical oncologists.

**Supplemental Table 6:** Change in RT boost recommendation by Radiation Oncologists after DCISionRT testing by DS ranges

|  |  | **Boost  Recommended** | | | **Pre- to Post-Test Change in Boost Recommended** | | **Total  Change in Boost Recommended** | | |
| --- | --- | --- | --- | --- | --- | --- | --- | --- | --- |
| **DS Range** | **n** | **Pre- Test (%)** | **Post-Test (%)** | **Net  Change (%)** | **Yes to No (%)** | **No to  Yes (%)** | **Overall  Change (%)** | **95% CI** | **p-Value** |
| **All *** | 1263 | 24% | 19% | -5% | 42% | 7% | 15% | 13-17% | <.0001 |
| DS <2 | 654 | 24% | 13% | -11% | 57% | 3% | 16% | 13-19% | <.0001 |
| DS 2-4 | 436 | 20% | 20% | 0% | 34% | 8% | 14% | 11-17% | 0.8964 |
| DS >4 | 173 | 33% | 41% | 8% | 12% | 18% | 16% | 11-22% | 0.0082 |

* Recommendations by radiation oncologists (independent) or tumor board

**Supplemental Table 5:** Impact of DCISionRT on Adjuvant Radiation Recommendations Stratified by Clinicopathologic Groups and DS Scores

|  |  | **RT Recommended** | | | **Pre- to Post-Test Change in RT Recommended** | | **Total  Change in RT Recommended** | | |
| --- | --- | --- | --- | --- | --- | --- | --- | --- | --- |
| **Clinical  Factor** | **n** | **Pre-Test (%)** | **Post-Test (%)** | **Net  Change (%)** | **Yes to No (%)** | **No to Yes (%)** | **Overall  Change (%)** | **95% CI** | **p-Value** |
| **All cases** | | | | | | | | | |
| All in group | 2007 | 71% | 51% | -20% | 41% | 31% | 38% | 36-40% | <0.0001 |
| DS <2 | 1026 | 72% | 29% | -43% | 63% | 7% | 47% | 44-50% | <0.0001 |
| DS 2-4 | 703 | 66% | 66% | 0% | 24% | 48% | 32% | 29-36% | 0.8946 |
| DS >4 | 278 | 80% | 92% | 12% | 4% | 75% | 18% | 14-23% | <0.0001 |
| **RTOG 9804-like^*^ Good Risk^#^** | | | | | | | | | |
| All in group | 1162 | 62% | 43% | -19% | 49% | 31% | 43% | 40-45% | <0.0001 |
| DS <2 | 658 | 66% | 23% | -43% | 69% | 6% | 48% | 44-51% | <0.0001 |
| DS 2-4 | 404 | 54% | 64% | 10% | 26% | 51% | 37% | 33-42% | 0.0015 |
| DS >4 | 100 | 65% | 93% | 28% | 2% | 83% | 30% | 22-40% | <0.0001 |
| **RTOG 9804-like^*^ Not Good Risk^#^** | | | | | | | | | |
| All in group | 832 | 84% | 61% | -23% | 33% | 30% | 32% | 29-36% | <0.0001 |
| DS <2 | 362 | 83% | 40% | -44% | 55% | 12% | 48% | 42-53% | <0.0001 |
| DS 2-4 | 294 | 83% | 70% | -13% | 23% | 39% | 26% | 21-31% | <0.0001 |
| DS >4 | 176 | 88% | 91% | 3% | 5% | 62% | 12% | 8-18% | 0.2752 |
| **ECOG E5194-like^**^ Low or Intermediate Grade Good Risk^#^** | | | | | | | | | |
| All in group | 1198 | 62% | 43% | -19% | 49% | 31% | 42% | 39-45% | <0.0001 |
| DS <2 | 676 | 66% | 23% | -43% | 69% | 6% | 47% | 43-51% | <0.0001 |
| DS 2-4 | 418 | 54% | 63% | 10% | 25% | 50% | 37% | 32-42% | 0.0013 |
| DS >4 | 104 | 65% | 93% | 28% | 1% | 83% | 30% | 22-39% | 0.0000 |
| **ECOG E5194-like^**^ High Grade Good Risk^#^** | | | | | | | | | |
| All in group | 362 | 83% | 55% | -28% | 38% | 22% | 36% | 31-41% | <0.0001 |
| DS <2 | 179 | 80% | 34% | -47% | 60% | 9% | 50% | 43-58% | <0.0001 |
| DS 2-4 | 123 | 81% | 67% | -14% | 25% | 35% | 27% | 20-35% | 0.0031 |
| DS >4 | 60 | 92% | 92% | 0% | 5% | 60% | 10% | 5-20% | 1.0000 |
| **ECOG E5194-like^**^ Not Good Risk^#^** | | | | | | | | | |
| All in group | 446 | 88% | 69% | -19% | 28% | 42% | 29% | 25-34% | <0.0001 |
| DS <2 | 170 | 89% | 49% | -40% | 48% | 21% | 45% | 37-52% | <0.0001 |
| DS 2-4 | 162 | 87% | 74% | -13% | 22% | 48% | 25% | 19-33% | 0.0010 |
| DS >4 | 114 | 87% | 90% | 4% | 5% | 60% | 12% | 7-20% | 0.2850 |

^*^ RTOG 9804-like “good risk” criteria is defined as low or intermediate grade, size ≤ 2.5 cm, negative margins (no-ink on tumor), non-palpable, screen detected.

^#^ Some patients have missing values.

**Supplemental Figure 1:** REMARK Diagram

**
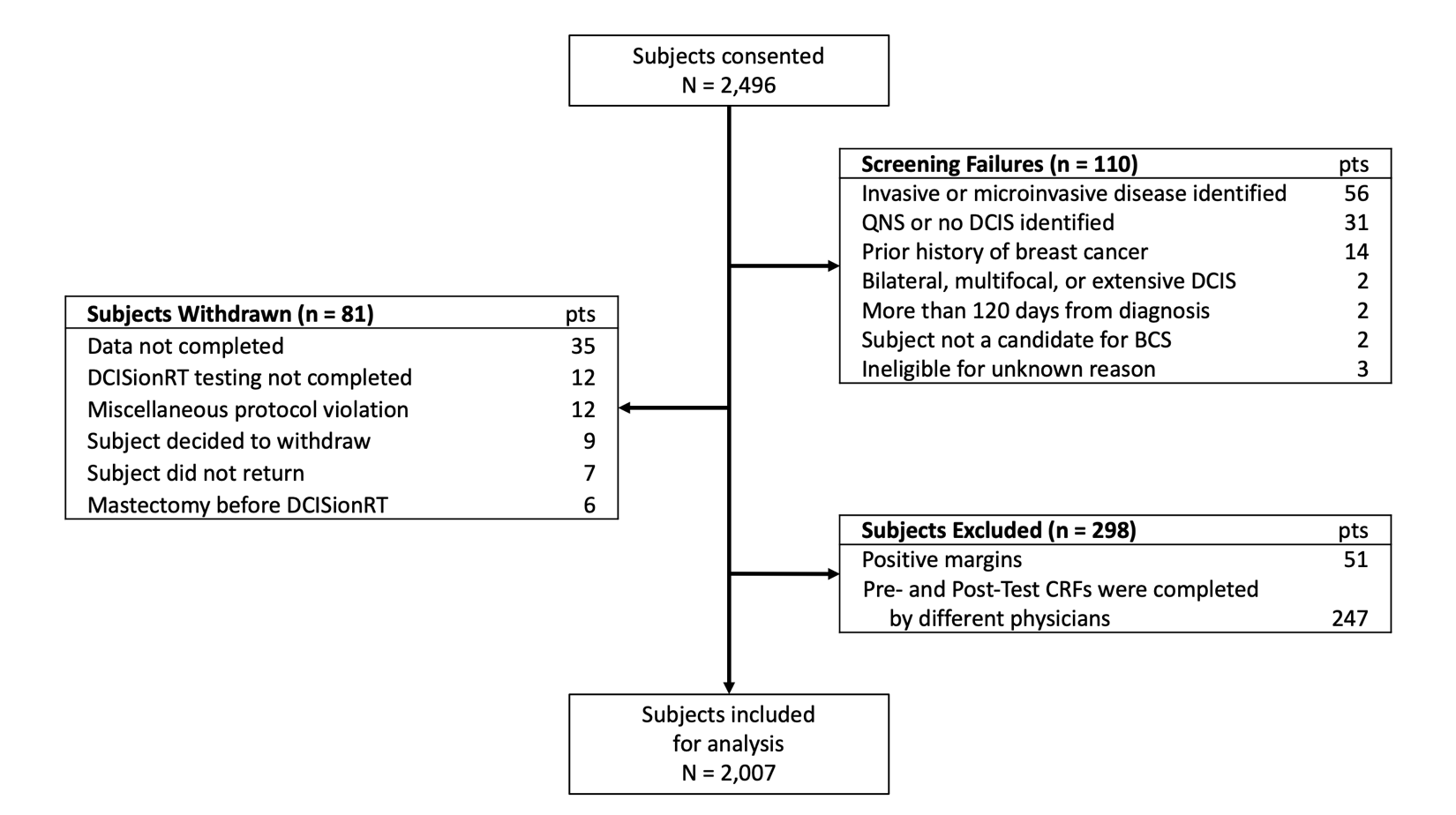
**

**Supplemental Figure 2:** Influence of continuous DS and patient preference on likelihood of recommendations for RT post-test reported by clinician specialty

1. Surgeons (independently)


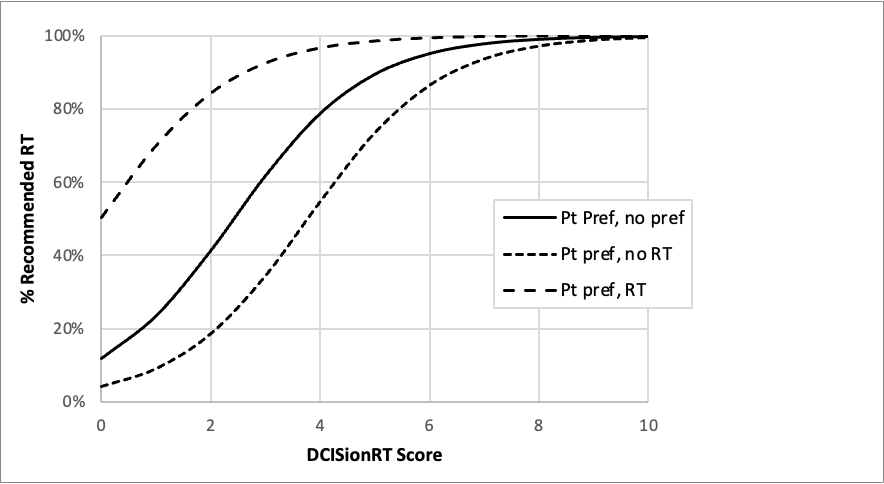


1. Radiation oncologists (independently) or tumor board


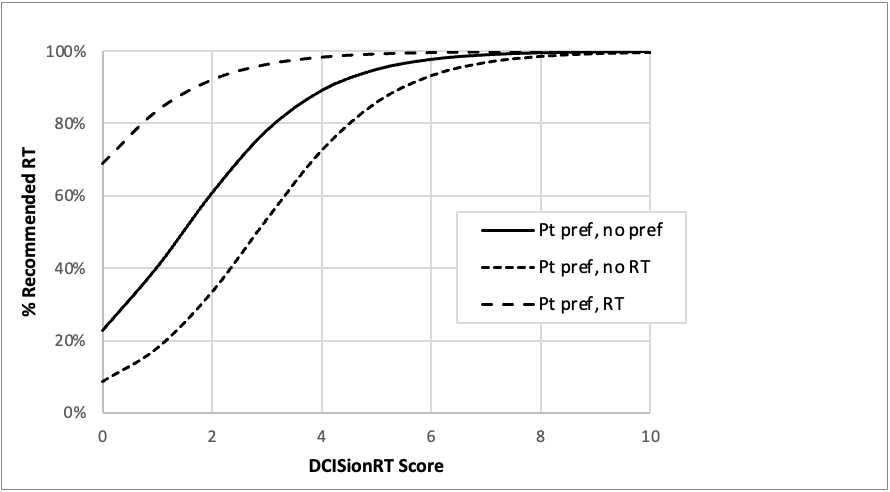


See [MVA Supplemental Table 4](#_Supplemental_Table_4:_1)

**Supplemental Figure 3:** Percentage of patients recommended RT with and without boost by DS ranges by radiation oncologists (independently) or tumor board


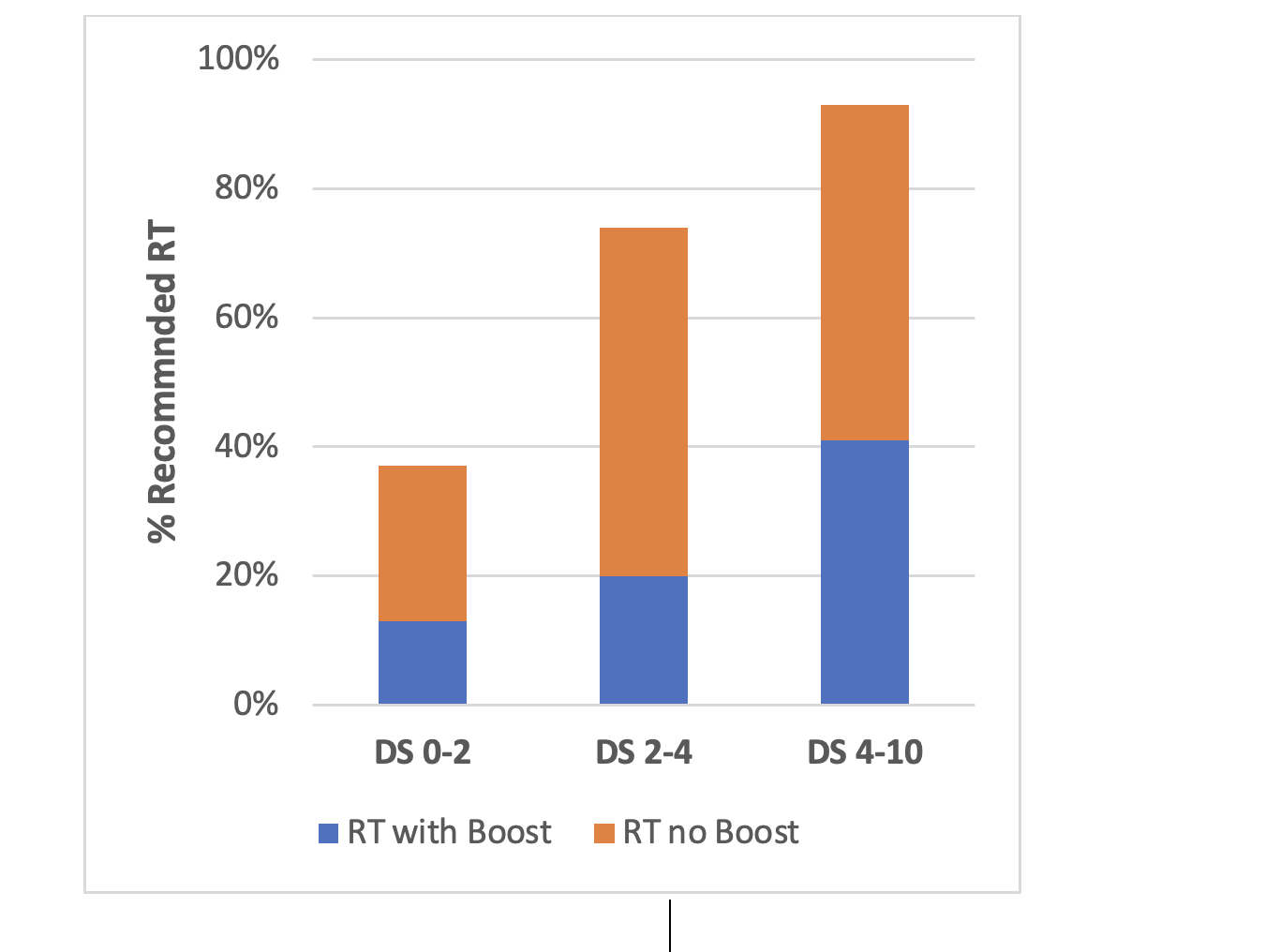

Supplement: Supplementary file 1 — Supplementary file1 (DOCX 579 kb) [file 10434_2024_15566_MOESM1_ESM.docx]
